# The completed mitochondrial genomes of *Globodera vulgaris* reveals new insights into the genus *Globodera* phylogeny

**DOI:** 10.1038/s41598-024-57736-1

**Published:** 2024-03-27

**Authors:** Sihua Yang, Huiying Zhu, Zaifu Yang, Xingyue Li, Yonglang Pan, Chunling Xu, Hui Xie

**Affiliations:** 1 Laboratory of Plant Nematology and Research Center of Nematodes of Plant Quarantine, Department of Plant Pathology, College of Plant Protection, South China Agricultural University, Guangzhou, 510642 People’s Republic of China; 2 Department of Plant Pathology, College of Agriculture, Guizhou University, Guiyang, 550025 People’s Republic of China; 3 Institute of Plant Protection, Sichuan Academy of Agricultural Sciences, Chengdu, 610066 People’s Republic of China

**Keywords:** *Globodera vulgaris*, *Globodera*, Mitochondrial genomes, Comparative mitochondrial genomics, Phylogenetic analysis, Evolution, Genetics, Microbiology

## Abstract

Due to the highly conserved structure, animal mitochondrial genome (mtDNA) is widely used in classification, evolution, phylogeny, population genetic structure and other fields. We reported on the five circle multipartite mtDNAs of a newly described species of *Globodera*, *Globodera vulgaris* (*Gv*) from potatoes in China. The results showed that the mtDNA of *Gv* was obtained through second- and third-generation sequencing, with a total length of 42,995 bp. It contained 12 protein-coding genes, two rRNA genes and 17 tRNA genes, which were distributed in different subgenomic circles. Comparison of the differences in mtDNA among *Gv*, *G*. *rostochiensis*, *G*. *pallida* and *G*. *ellingtonae* showed that the size and arrangement of the genes in the mtDNA of the genus *Globodera* were variable and not conserved. The codon usage bias of the mitochondrial protein-coding gene of *Gv* showed that *Gv* might have originated from locally and more primitive group of existing *Globodera*. Based on the cytochrome c oxidase subunits I genes (*COX1*) and the nicotinamide adenine dinucleotide dehydrogenase subunits I genes (*ND1*), and the results showed that *Gv* was clustered with *Globodera* spp. according to the *COX1* and *ND1* in scmtDNA-V, while *Gv* was clustered with Meloidogyne spp. according to *ND1* in scmtDNA-III. The results of this study provided a new basis for understanding the multipartite structure of mtDNA as a phylogenetic and taxonomic feature of the genus *Globodera*. The number of subgenomic circles is a diagnostic feature of species and the arrangement order and size of mitochondrial protein-coding genes also have important application value in species identification within the genus.

## Introdution

The potato cyst nematodes (PCN), including *Globodera rostochiensis* and *G*. *pallida*, originates in South America and are the most harmful plant-parasitic nematode of potato yield worldwide^1^. PCN has been reported in most potato-producing regions of Europe, Africa, Asia, North, Central and South America and Oceania^2^. According to statistics, PCN has caused 10–12% yield loss in global potato production^3,4^ and more than 60% yield loss, as high as 80–90% or even no harvest when the disease is serious in potato-planted areas^1,5–9^. PCN continues to be treated as a quarantine pest by regional plant protection organisations worldwide^10^.

Nematodes are multicellular eukaryotes. Mitochondria exist in almost all known eukaryotes^11^ and contain a set of genomes independent of the nucleus, namely, the mitochondrial genome (mtDNA). Compared to nuclear genes, mtDNA has the characteristics of smaller size, faster evolution rate, and matrilineal inheritance, which makes it widely used in classification, evolution, phylogeny, population genetic structure and other fields^12–14^. It is also an effective molecular target for the classification and identification of nematodes^15^. Plant-parasitic nematodes, whose mtDNA sequencing had been completed, are economically important species, including *G*. *rostochiensis*, *G*. *pallida*, *G*. *ellingtonae*, *Aphelenchoides besseyi*, *A*. *medicagus*, *Bursaphelenchus xylophilus*, *B*. *mucronatus*, *Heterodera glycines*, *Meloidogyne incognita*, *M*. *javanica*, *M*. *arenaria*, *M*. *enterolobii*, *M*. *graminicola*, *M*. *chitwoodi*, *Xiphinema americanum* and *Hoplolaimus columbus*^16–28^.

The mtDNA structure in metazoans is generally a highly conserved single cyclic molecule^29^ that contains a specific set of genes whose order is highly conserved throughout the phylum^30^. However, among the reported mtDNAs of plant nematodes, those of several species of the genus *Globodera* are multipartite, whereas those of other genera are monopartite^16–28^. The first metazoan reported to have multipartite mtDNA circles is *G*. *pallida*, which has at least six subgenomic mitochondrial circles (scmtDNAs), approximately 6–9 kb in size^16,18^. Subsequently, *G*. *rostochiensis* was reported to contain at least seven scmtDNAs of similar sizes^19,31^, whereas *G*. *ellingtonae* contains two scmtDNAs, 17,757 and 14,365 bp in size^26^.

At present, *COX1* gene in the mtDNA of nematodes has attracted much attention for its use in the identification of related species^32^, and for resolution at lower taxonomic levels such as species and subspecies groups^33–35^. Ohki et al.^36^ showed that the *G*. *pallida* population from Japan was close to the populations from Europe and North America, compared the cytochrome b gene (*CYTB*). Picard et al.^37^ and Plantard et al.^38^ confirmed that *G*. *pallida* was introduced into Europe from North America then spread to various parts of the world by combining its phylogeography and phylogenetic analysis of *CYTB*. Subbotin et al.^35^ collected 148 populations of *Globodera* species from 23 countries and conducted a phylogenetic analysis of cytochrome c oxidase subunits I gene (*COX1*) and *CYTB* genes and the internal transcribed spacers of ribosomal DNA (ITS). The analysis depicted that *Globodera* species are mainly divided into two branches: one branch from South America and North America, which mainly parasitizes *Solanaceae* plants, and the second branch from Africa, Europe and New Zealand, which parasitizes *Asteraceae* and other plants^35^. These studies indicated that mtDNA data could not only construct an effective framework for the phylogeny of nematodes in higher order but also effectively evaluated the genetic variation among related species to explore their genetic relationship and taxonomic status.

One species of *Globodera* was successively found in the potato fields of Guizhou, Sichuan and Yunnan provinces in China in 2018. Jiang et al.^39^, Gu et al.^40^ and Peng et al.^41^ identified this species as *G*. *rostochiensis* mainly based on its ITS sequences. However, Xu et al.^42^ found that although the ITS sequence of this species was very similar to that of *G*. *rostochiensis*, its morphological characteristics, such as the obvious vela on the spicules of males, were substantially different from those of *G*. *rostochiensis*. Moreover, they were substantially different in the host range, pathogenicity, field damage and symptom performance. Therefore, this *Globodera* species was identified as a new species, named as *Globodera vulgaris* (*Gv*)^42^. In the present study, the mtDNA of *Gv* collected from the potato fields in Guizhou, was sequenced and analysed. Based on the sequencing results, the origin and phylogenetic relationship of *Gv* were analysed and established, and the role of the mitochondrial genome in the classification and identification of the *Globodera* genus was discussed.

## Results

### The mtDNA assembly and PCR verification after high-throughput sequencing

**Figure 1:**
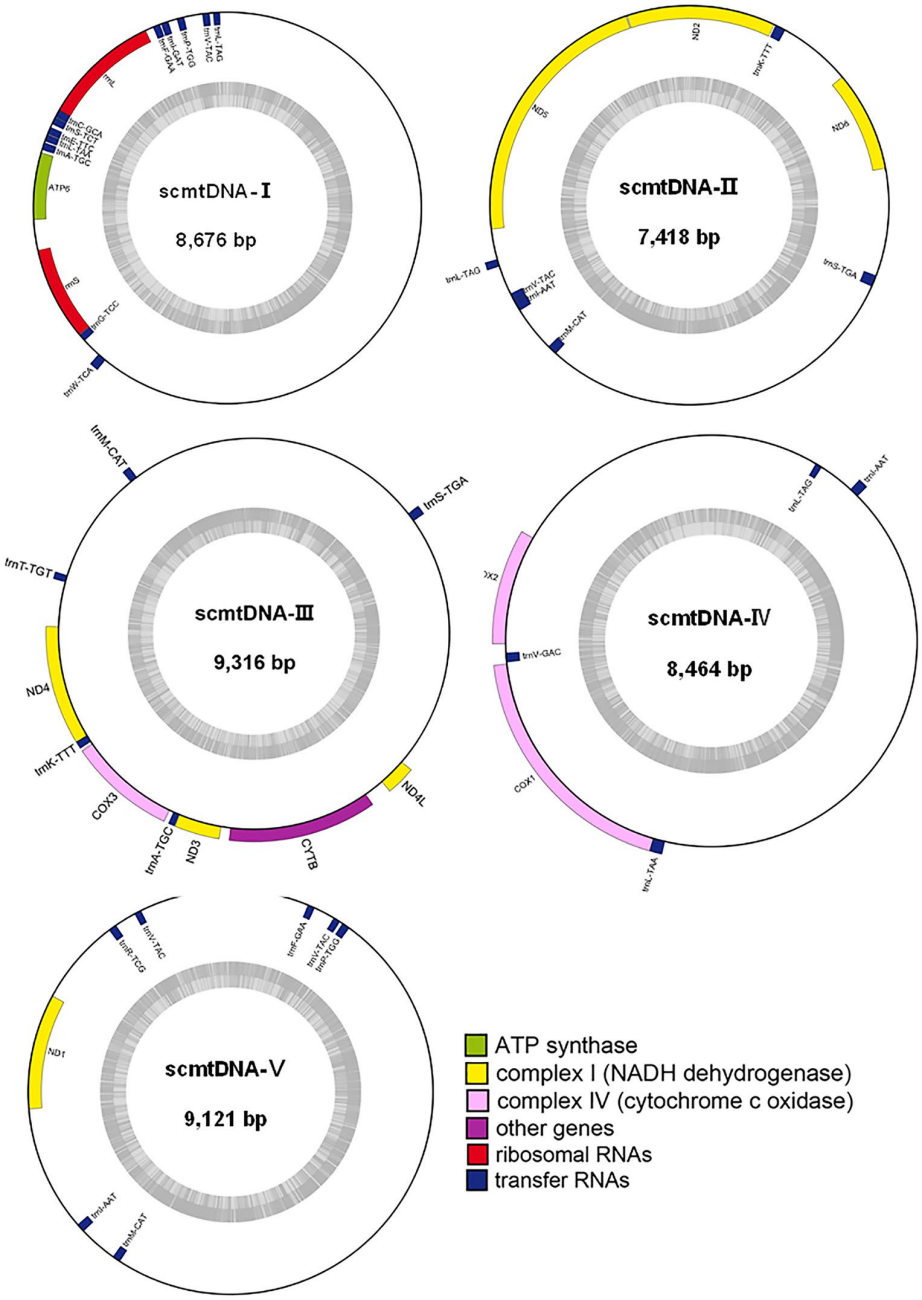
Annotation and structure of subgenomic mitochondrial circles (scmtDNA) in *Globodera vulgaris*. The transcription direction of the in-circle genes is clockwise, whereas that of the out-circle genes is the opposite. Different functional genes were identified using different colours. The built-in dark grey histogram show the GC content of the genome, and the middle grey line represent the 50% threshold.

The mtDNA of *Gv* was assembled, and a 42,995-bp mtDNA was obtained, which was assembled into five scmtDNAs with a size of 7–9 kb and named scmtDNA-I to V (GenBank PP407208-PP407212) (Fig. 1). A total of 31 genes were encoded in the whole mtDNA, including 12 protein-encoding genes, namely, one ATP synthase subunits gene (*ATP6*), three cytochrome c oxidase subunits genes (*COX1*, *COX2* and *COX3*), one cytochrome b gene (*CYTB*), seven nicotinamide adenine dinucleotide (NADH) dehydrogenase subunits genes (*ND1*, *ND2*, *ND3*, *ND4*, *ND4L*, *ND5* and *ND6*), two ribosomal RNA (*rrnL* and *rrnS*) and 17 transporter RNA genes (tRNAs). The A + T content of each scmtDNA sample ranged from 63.2 to 69.2%, indicating a significant AT bias. No gene overlap was detected in the scmtDNAs, and gene spacer regions were present. To verify the assembly results, specific fragments of scmtDNA-I to -V were successively amplified with the corresponding primers using total DNA as the template and amplified fragments of approximately 3 kb. The sequences were determined to be 3096 and 2884 bp, 3063 and 2961 bp, 3042 and 3006 bp, 3016 and 2943 bp, 2987 and 2915 bp in size, respectively, which were consistent with the estimated sizes (S1 Fig.).

### Protein-encoding genes in the mtDNA of *Gv*

Twelve protein-encoding genes were annotated in the mtDNA of *Gv*. All protein-encoding genes had a complete initiation codon, whereas two genes (*COX1* and *ND1*) had an undetermined termination codon (Table 1). The codons of the protein-encoding gene in mtDNA of *Gv* showed significant AT bias, and most of them exhibited significant AT negative skew (0.56–0.39) and GC positive skew (0.29–0.65) (Table 1).

**Table 1:** Nucleotide composition and bias of protein-encoding genes in the mitochondrial genome of *Globodera vulgaris.*

Genes	Nucleotide composition				A + T content	AT skew	GC skew	Initiation codon	Termination codon
	A	T	G	C					
*ATP6*	54.4	15.0	8.3	22.9	69.4	0.57	− 0.47	ATA	TAA
*COX1*	19.0	43.1	24.4	13.5	62.1	− 0.39	0.29	ATT	–
*COX2*	16.7	49.7	24.0	9.6	66.4	− 0.50	0.43	ATG	TAG
*COX3*	17.0	50.5	21.6	11.0	67.4	− 0.50	0.33	ATA	TAA
*CYTB*	19.5	51.2	19.4	10.0	70.6	− 0.45	0.32	ATA	TAG
*ND1*	57.8	14.0	8.7	19.6	71.8	0.61	− 0.39	ATA	–
*ND2*	55.5	17.7	7.3	19.5	73.2	0.52	− 0.46	ATA	TAG
*ND3*	17.6	58.2	20.0	4.2	75.8	− 0.54	0.65	TTG	TAG
*ND4*	17.3	54.8	19.2	8.8	72.1	− 0.52	0.37	ATT	TAA
*ND4L*	16.9	60.0	19.1	4.0	76.9	− 0.56	0.65	ATT	TAG
*ND5*	53.5	18.1	8.9	19.5	71.6	0.50	− 0.37	ATT	TAA
*ND6*	53.4	16.1	10.7	19.9	69.5	0.54	− 0.30	ATG	TAA

### The tRNAs and rRNAs of the mtDNA of *Gv*

**Figure 2:**
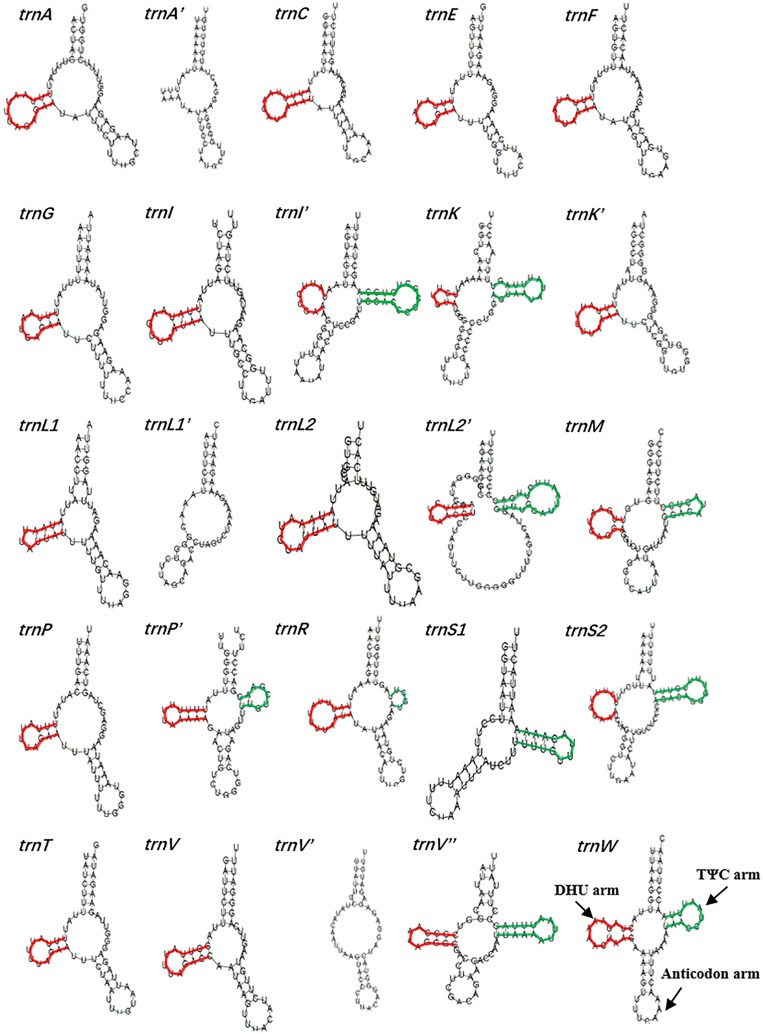
The secondary structure prediction of tRNAs in the mitochondrial genome of *Globodera vulgaris trnA*: *trnA* in subgenomic mitochondrial circle I of *G. vulgaris* (scmtDNA-I); *trnA′*: *trnA* in scmtDNA-III; *trnC*: *trnC* in scmtDNA-I; *trnE*: *trnE* in scmtDNA-I; *trnF*: *trnF* in scmtDNA-I; *trnG*: *trnG* in scmtDNA-I; *trnI*: *trnI* in scmtDNA-II; *trnI′*: *trnI* in scmtDNA-IV; *trnK*: *trnK* in scmtDNA-II; *trnK′*: *trnK* in scmtDNA-III; *trnL1*: *trnL1* in scmtDNA-II; *trnL1′*: *trnL1* in scmtDNA-IV; *trnL2*: *trnL2* in scmtDNA-I; *trnL2′*: *trnL2* in scmtDNA-IV; *trnM*: *trnM* in scmtDNA-II, scmtDNA-III and scmtDNA-V; *trnP*: *trnP* in scmtDNA-I; *trnP′*: *trnP* in scmtDNA-V; *trnR*: *trnR* in scmtDNA-V; *trnS1*: *trnS1* in scmtDNA-I; *trnS2*: *trnS2* in scmtDNA-II and scmtDNA-III; *trnT*: *trnT* in scmtDNA-III; *trnV*: *trnV* in scmtDNA-I; *trnV′*: *trnV* in scmtDNA-II; *trnV′′*: *trnV* in scmtDNA-IV; *trnW*: *trnW* in scmtDNA-I.

The MITOS WebServer online tool was used to predict tRNA in the mtDNA of *Gv*, and a total of 17 tRNAs with a length range of 46–79 bp were identified, 11 (*trnA*, *trnE*, *trnF*, *trnI*, *trnK*, *trnL1*, *trnL2*, *trnM*, *trnP*, *trnS* and *trnV*) of which were multiple copies in different scmtDNAs and had different secondary structures (Fig. 2). The secondary structures of most of the tRNAs were typical clover structures (including TΨC arm, DHU arm and anticodon arm), 11 of which lacked TΨC arms. In addition, *trnA* in scmtDNA-III(*trnA′*) lacked DHU rings, and *trnV* in scmtDNA-II (*trnV″*) lacked DHU arms. The remaining *trnI* in scmtDNA-IV and scmtDNA-IV (*trnI′*), *trnK* in scmtDNA-III, *trnL2* in scmtDNA-IV (*trnL2′*), *trnM* in scmtDNA-II, scmtDNA-III and scmtDNA-V, *trnP* in scmtDNA-IV (*trnP′*), *trnR* in scmtDNA-IV, *trnS2* in scmtDNA-II and scmtDNA-III, *trnV* in scmtDNA-IV (*trnV″*) and *trnW* in scmtDNA-I present a complete clover structure. The rRNA in the mtDNA of *Gv* was obtained by comparison with that of similar species. The sizes of *rrnL* and *rrnS* were 846 and 673 bp, respectively, which were not different from the rRNA lengths of other plant-parasitic nematodes, and all had high A + T content.

### Non-coding regions in the mtDNA of *Gv*

The mtDNA of *Gv* consisted of 46 non-coding regions of variable sizes, ranging in length from 1 to 5327 bp. Each scmtDNA of *Gv* had a long non-coding region with lengths of 5327, 2223, 2297, 3472 and 4490 bp, located between *trnW* and *trnL* in scmtDNA-I, *trnM* and *trnS* in scmtDNA-II, *trnS* and *trnM* in scmtDNA-III, *trnL* and *trnI* in scmtDNA-IV, and *tRNA* and *trnP* in scmtDNA-V, with AT contents of 65.3%, 59.8%, 59.9%, 60.0%, and 59.4%, respectively. The circles shared a high sequence identity region, in their longest non-coding regions. There were 98% identical sites in the ~ 2.3 kb shared sequence region between mtDNA-III position 983–2297 and mtDNA-II position 4700–6914 and mtDNA-V position 6002–8218.

### Comparison of the mtDNA of four species of the genus *Globodera*

The mtDNA of *Gv* obtained in this study was compared to that of three other species of the genus *Globodera*. The results (Table 2) showed that the length of the mtDNA of *Gv* was close to that of *G*. *rostochiensis* and *G*. *pallida*, with lengths of 42,995, 4160 and 45,071 bp, respectively, but different from the number of scmtDNAs, with five, seven and six, respectively. The mtDNA of *G*. *ellingtonae* was the shortest, with a length of 32,122 bp and only two subgenomic circles.

**Table 2:** Comparison of the mitochondrial genomes (mtDNA) of *Globodera* nematodes.

Species	The number of subgenomic circle of mtDNA	Genome length/bp	The length of subgenomic circle of mtDNA/bp	GenBank
*G*.* vulgaris*	5	42,995	I 8676	
			II 7418	
			III 9316	
			IV 8464	
			V 9121	
*G*. *rostochiensis*^19,31^	7	41,601	I 9210	EF193005
			II 6604	EF462976
			III 3192	EF462977
			IV 6010	EF462978
			V 5480	EF462979
			VI 5797	EF462980
			VII 5308	EF462981
*G*. *pallida*^16,18^	6	45,071	I 9428	AJ249395
			II 7511	DQ631911
			III 6400	DQ631912
			IV 7560	DQ631913
			V 7472	DQ631914
			VI ~ 6700	Sequencing is not complete
*G*. *ellingtonae*^26^	2	32,122	I 17,757	KU726971
			II 14,367	KU726972

The A + T content, AT skew and GC skew were used to measure differences in the base composition. AT skew and GC skew were used to describe the difference in the contents of A and T, and the difference in the contents of G and C, respectively^43^. The A + T content in the mitochondrial genomes of the four species of *Globodera* was between 65.6 and 67.0% (Table 3), with an average of approximately 65%, showing a significant AT bias, and the differences between species were not significant. However, *Gv* mitochondrial genome showed positive AT skew and negative GC skew, whereas the other three species showed significantly negative AT skew and positive GC skew, indicating that the base composition of *Gv* mitochondrial genome was different from that of other species of *Globodera*, and the nucleotide usage of *Gv* was more inclined to A and C.

**Table 3:** Base content (%), AT skew and GC skew of the mitochondrial genome of *Globodera.*

Species	A%	T%	G%	C%	A + T	AT skew	GC skew	References
*G*.* vulgaris*	34.8	31.2	16.2	17.9	65.9	0.05	− 0.05	
*G*.* rostochiensis*	20.4	46.4	20.6	12.6	66.7	− 0.39	0.24	Gibson et al. and Riepsamen et al.^19,31^
*G*.* pallida*	21.9	43.7	20.6	13.8	65.6	− 0.33	0.20	Gibson et al. and Armstrong et al.^16,18^
*G*.* ellingtonae*	20.8	46.3	21.6	11.4	67.0	− 0.38	0.31	Phillips et al.^26^

### Comparison of protein encoding genes in the mtDNA of four *Globodera* species

Compared to *Gv*, *G*. *rostochiensis* lacks *ND2* and *ND6*, whereas *G*. *pallida* lacks *ND2*, *ND4L* and *ND5*, and *ATP6* with incomplete sequences^16,18,19,26,31^. There were eight protein-coding genes in the mtDNA of these four species of *Globodera*, namely *ATP6*, *COX1*, *COX2*, *COX3*, *CYTB*, *ND1*, *ND3* and *ND4*. However, there were some differences in gene length and similarity (Table 4). The lengths of *COX3*, *CYTB* and *ND3* were similar among the four species, whereas those of *ATP6*, *COX1*, *COX2*, *ND4* and *ND1* varied from 45 to 511 bp. The results of the gene similarity comparison showed that the *ATP6*, *COX1*, *COX2*, *ND1* genes of *Gv* were quite different from those of the other three species. At the nucleotide level, the most differentially expressed gene was the *ND6*, and the least differentially expressed one was *CYTB*. Most of the protein-encoding genes of *Gv* had the highest similarity with those of *G*. *rostochiensis*, but the lowest similarity with those of *G*. *pallida*. In conclusion, significant differences are present in the category, size and sequence similarity of the protein-encoding genes in the mitochondrial genomes of the different species of *Globodera*.

**Table 4:** Comparison of the length (bp) and similarity of the mitochondrial protein-encoding genes in *Globodera*.

Genes	*G*.* vulgaris*	*G*. *rostochiensis*^19,31^		*G*. *pallida*^16,18^		*G*. *ellingtonae*^26^	
	Gene length	Gene length	Similarity ^a^	Gene length	Similarity ^a^	Gene length	Similarity ^a^ (%)
*ATP6*	480	411	76.30%	Incomplete		588	81.46
*COX1*	1582	1389	75.52%	1389	75.59%	1509	75.49
*COX2*	726	681	81.40%	681	81.10%	675	83.95
*COX3*	783	783	99.49%	773	72.54%	774	81.52
*CYTB*	1083	1084	99.82%	1089	74.33%	1074	81.66
*ND1*	843	657	74.35%	830	74.70%	830	78.73
*ND2*	897	No reports		No reports		810	78.50
*ND3*	330	366	99.70%	336	70.52%	306	77.45
*ND4*	1434	1212	99.42%	1208	77.34%	1206	81.31
*ND4L*	225	261	98.67%	No reports		228	83.44
*ND5*	1617	609	78.16%	No reports		1515	78.80
*ND6*	606	No reports		442	65.21%	402	72.15

Superscript a indicates the sequence similarity of the mitochondrial protein-encoding genes of *Globodera vulgaris* to those of the three species of *Globodera.*

### Codon usage bias of the mtDNA of* Globodera* species

ENC refers to the number of effective codons used in a gene and is a quantitative value of the bias of the codon usage frequency of a gene from the average usage frequency of synonymous codons, which is often used to reflect the bias in codon usage. The smaller the ENC value, the stronger the codon usage^44^. A gene with an ENC value lower than 35 is generally considered to have an obvious codon usage bias^45^. The results of the ENC value calculation and analysis of the four species of *Globodera* showed that the ENC values of the protein-encoding genes for *Gv*, *G*. *rostochiensis*, *G*. *pallida* and *G*. *ellingtonae* were 44.1 (28.8–52.8), 41.2 (35.8–47.1), 43.6 (38.8–47.7) and 39.1 (32.1–48.2), respectively (Table 5). The ENC values of *COX1*, *COX2*, *CYTB* and *ND4L* in *Gv* were the lowest among the four species, which indicated that *Gv* had a relatively strong codon usage bias in these genes, whereas the ENC values of *ATP6*, *COX3*, *ND2*, *ND4*, *ND5* and *ND6* were the highest, indicating that the codon usage of those genes was relatively weak.

**Table 5:** The effective number of codons (ENC) of the mitochondrial protein-encoding genes in *Globodera* species.

Protein coding gene	*G*.* vulgaris*	*G*. *rostochiensis*^19,31^	*G*. *pallida*^16,18^	*G*. *ellingtonae*^26^
*ATP6*	52.8	43.8	Incomplete	33.9
*COX1*	44.6	47.1	47	48.1
*COX2*	39	42.7	43.1	48.2
*COX3*	44.8	44.3	42.4	36.7
*CYTB*	40	44.6	39.8	37.9
*ND1*	46.1	38	47.7	46.3
*ND2*	50	–	–	36.6
*ND3*	36.4	36	43.9	35.8
*ND4*	48.4	35.8	38.8	34.9
*ND4L*	28.8	36.4	–	32.1
*ND5*	50.7	43.1	–	42.2
*ND6*	47.9	–	45.7	36.2

### Phylogenetic analysis

**Figure 3:**
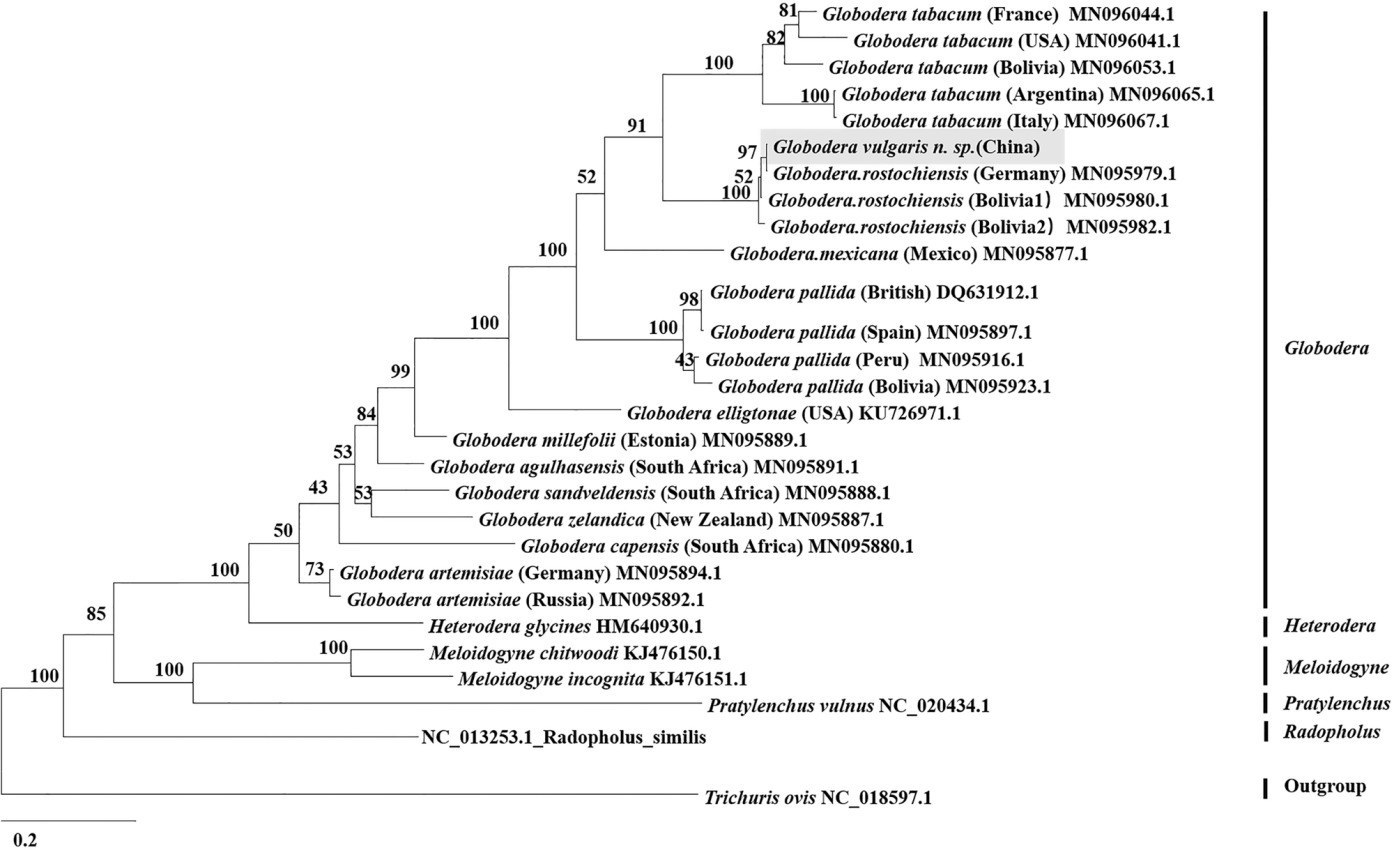
The phylogenetic tree inferred from the Bayesian method (BI) based on COX1 sequences.

**Figure 4:**
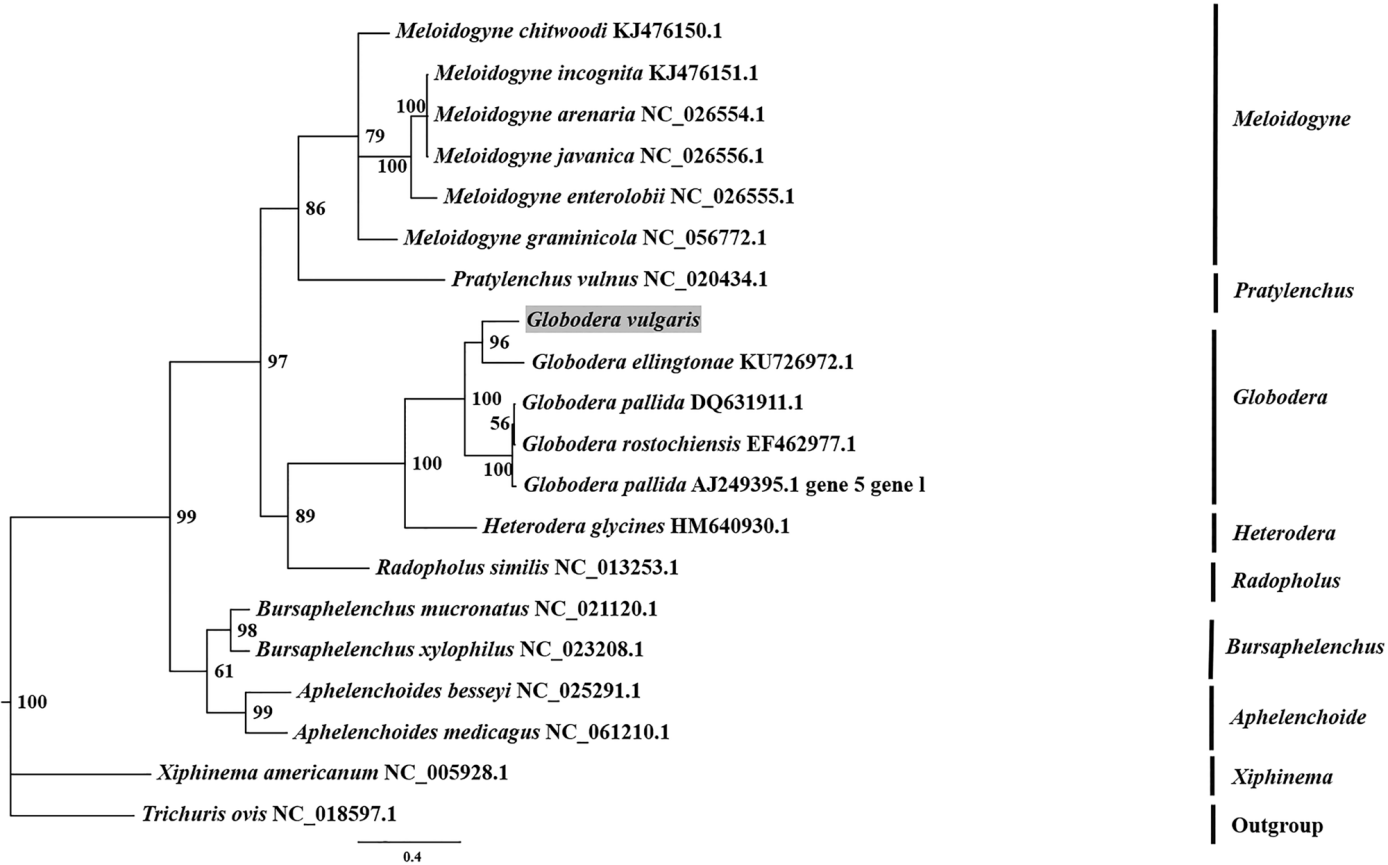
The phylogenetic tree inferred from the Bayesian method (BI) based on ND1 sequences.

The phylogenetic tree was constructed based on the *COX1* gene of *Gv* and other 27 species (or groups) of Tylenchida, with *Trichuris ovis* as the outgroup (Fig. 3). *Globodera* spp., *H*. *glycines*, *M*. *chitwoodi*,* M*. *incognita*, *Pratylenchus vulnus* and *Radopholus similis* were clustered in separate branches. The closest relationships with *Globodera* spp. were with *H*. *glycines*, followed by *M*. *chitwoodi*,* M*. *incognita*,* P*. *vulnus* and *R*. *similis*. Within this branch of *Globodera* spp., different geographical populations were clustered into separate branches. *Gv* had the closest relationship with *G*. *rostochiensis*, particularly in the German population, followed by *G*. *tabacum*. The phylogenetic tree was constructed based on the *ND1* gene of *Gv* and other 18 species, with *Trichuris ovis* as the outgroup (Fig. 4). Aphelenchida and Tylenchida were clustered in separate branches. The *ND1* gene in scmtDNA-V had the closest relationship with *G. ellingtonae* and clustered with other *Globodera* spp. as well as *Radopholus similis* into a branch. The experimental results showed that different target genes constructed different phylogenetic trees. Both of the sequences of *COX1* and *ND1* in scmtDNA-V were clustered with *Globodera* spp., but there were differences in the closest species.

## Discussion

In metazoans, mtDNA generally has a single circular structure, and the division of mtDNA into multiple particles is a rare phenomenon^29^. Currently, the majority of mtDNA from plant-parasitic nematodes that have complete mtDNA sequencing have a single circular structure, except for species of the genus *Globodera*^16–28^. The mtDNAs of the three species of *Globodera* reported were multipartite structures with different scmtDNA numbers. *G. rostochiensis* had at least seven scmtDNAs, whereas *G*. *pallida* had six, and *G*. *ellingtonae* had two^16,18,18,26,31^. This study showed that *Gv* had five scmtDNAs, and that its mtDNA size was different from that of the other three species of *Globodera*. Therefore, it was speculated that the multipartite structure of mtDNA was an important phylogenetic feature and important basis for determining the taxonomic status of the genus *Globodera*, distinguishing it from other genera. The size of mtDNA and number of scmtDNAs were the important characteristics for species identification and differentiation in the genus *Globodera*.

The order of mitochondrial genes in metazoans is generally conserved within the same genus or even within the same order^46^. However, a comparison of mitochondrial protein-coding genes among the four species of *Globodera* revealed obvious differences in arrangement order and size. Therefore, mitochondrial genes might have important application value in species identification of the genus *Globodera*.

Seventeen tRNAs were annotated, and five tRNAs (*trnD*, *trnH*, *trnN*, *trnQ* and *trnY*) that exist in other nematodes were missing from the mtDNA of *Gv*. The *trnS1*and *trnH* were absent in *G*. *rostochiensis* and *G*. *pallida*^16,18,19,31^. Multiple copies of *trnA*, *trnE*, *trnF*, *trnI*, *trnK*, *trnL1*, *trnL2*, *trnM*, *trnP*, *trnS* and *trnV* occurred in the mtDNA of *Gv*, whereas multiple copies of *trnD*, *trnE*, *trnI*, *trnK*, *trnP*, *trnQ*, *trnT* and *trnY* in the mtDNA of *G*. *rostochiensis*, and *trnD*, *trnH*, *trnI*, *trnK*, *trnP*, *trnT* and *trnV* in the scmtDNA of *G*. *pallida* were also observed^16,18,19,31^. No multiple copies of tRNA were found in the mtDNA of *G*. *ellingtonae*^26^. It was reported that *trnS2* and *trnV* were two copies in the mtDNA of two populations of *M*. *graminicola*, with substantial differences between the two copies^25^. It has been speculated that copies of *trnS2* and *trnV* may have independently originated or may have been “involved” in the evolution of other exogenous tRNAs ^47–49^. The mtDNAs of *Gv*, *G*. *rostochiensis*, *G*. *pallida* and *G*. *ellingtonae* have multipartite structures with many scmtDNAs, suggesting that the multi-copy phenomenon of tRNA in the mtDNA of *Globodera* might be the result of gene recombination between the subgenomes. However, the multiple copies of some tRNA genes in *Gv* were quite different among subgenomes, which were not found in *G*. *rostochiensis* and *G*. *pallida*, and might be the result of exogenous tRNA intervention. In addition, except for the fact that eight tRNAs of *Trichinella spiralis* could be folded into a typical clover structure^50^, no tRNAs of other nematodes have been reported as having clover structures, including *G*. *ellingtonae*, where none of their 22 tRNA structures had a typical clover structure^26,51^, and the tRNAs structures of *G*. *rostochiensis* and *G*. *pallida* have not been reported. In this study, it was demonstrated that nine tRNAs (*trnI*′, *trnK*, *trnL2*′, *trnM*, *trnP′*, *trnR*, *trnS2*, *trnV″*, and *trnW*) of *Gv* had folded into the complete clover structure. Thus, the tRNA in the mtDNA of *Gv* was quite different from that in other nematodes, including *G*. *rostochiensis*, *G*. *pallida* and *G*. *ellingtonae*. Nematode mitochondrial genomes are known to exhibit a strong nucleotide compositional bias^52^. The base composition bias of mtDNA was mainly caused by the asymmetric mutation and selection pressure of four bases (A, T, G and C), which were mainly owing to DNA replication and gene transcription; In other words, mitochondrial genes in some species undergo frequent rearrangements, mutations and selection pressure during replication and transcription, resulting in a biased reversal of mitochondrial base composition; that is, the base composition changes from A and C–T and G^53^. The nucleotide skew is attributed to mutation pressure and selection pressure^54^. Interestingly, closely related species that have evolved in significantly different environments demonstrate similar base usage strategies. For instance, one species may exhibit an excess of thymine (T) over adenine (A), while a closely related species may show a bias towards using A over T^55^. In the case of the mtDNA of *Gv* and three other species of *Globodera*, it was observed that *Gv* displayed AT-positive and GC-negative skew, whereas the other three species showed AT-negative and GC-positive skew. This suggests that *Gv* may have adopted a unique adaptive evolutionary strategy.

Methionine-encoding ATG is commonly used as the initiation codon for translation in the nuclear genome; however, owing to obvious AT bias, the genetic code of mtDNA in nematodes mainly consists of AT. The initiation codons of protein-encoding genes in nematodes are ATT and TTG, ATA, ATC, CTT, GTT and GTG. TAA and TAG have been used as transcription termination signals in most nematodes^56^. Five sets of initiation codons were used in the mtDNA of *Gv*, among which ATA was used six times; ATT, ATG and TTG were used twice; and ATC was used once. The stop codons of two genes (*COX1* and *mt5-ND1*) could not be determined, whereas TAG was used four times, and TAA was used seven times for genes with clear stop codons. In *G*. *ellingtonae*, the initiation codon TTG was used eight times, whereas TTA was used twice, and ATA and ATT were used once. The stop codons of three protein-encoding genes (*COX1*, *COX2* and *ND1*) could not be determined, whereas TAG was used seven times, and TAA was used twice for genes with clear stop codons^26^. Therefore, differences were present in the codon usage rules of protein-coding genes in the mtDNAs of *Gv* and *G*. *ellingtonae*, although both had an obvious AT bias. The codon usage of protein-encoding genes in the mtDNA of *G*. *rostochiensis* and *G*. *pallida* has not yet been analysed and reported.

Generally, different species have different codon usage patterns, and genes of the same species may adopt similar codon usage strategies^57^. Species with closer phylogenetic relationships or more similar living environments might have similar codon usage patterns^58^, which might be the result of natural selection and species adaptation and widespread in organisms, reflecting some evolutionary phenomena^59,60^. Genes with higher codon usage bias tend to have higher expression levels^58^. The codon usage bias of the mitochondrial gene of *Gv* was weaker than that of the other three species of *Globodera*, indicating that its gene expression and evolution levels were lower and suggesting that *Gv* might be a more primitive group.

The base composition and codon usage of mtDNA of *Gv* differed from those of the other three species of *Globodera*, with more primitive characteristics in base selection and codon bias. In addition, the results of field investigations and experimental tests have shown that *Gv* could parasitize not only Solanaceae plants, such as potatoes and tomatoes, but also many other families, such as Caryophyllaceae, Asteraceae and Amaranthaceae^42^. Therefore, we speculated that *Gv* was an indigenous and more primitive group than *G*. *rostochiensis*. *Gv* might initially parasitize a variety of weeds. With the introduction of potato into China and its large-scale cultivation, potato has become its dominant host, and its characteristics, which are similar to those of *G*. *rostochiensis* might be the result of host adaptability.

## Conclusion

In this study, the mtDNA of *Gv* was assembled, as well as the comparison of its mtDNA with those of other plant nematodes, revealed that the multipartite structure of mtDNA was the phylogenetic feature of *Globodera* spp. and the taxonomic feature of the genus. The number of scmtDNA was the distinguishing feature of the species in the genus, and the number, arrangement and size of mitochondrial protein-encoding genes have important application value in species identification for the genus *Globodera*. The regularity of codon usage and base composition bias of mitochondrial genes might also hold significant value for studying the origin and phylogenetic relationships of *Globodera*. Furthermore, the number, variety, and structure of tRNAs in the mitochondrial genome might have application value for classification, origin, and phylogenetic studies in the *Globodera* genus. This study revealed that *Gv* bases composition were biased towards A and C without bias inversion, and mitochondrial gene codon usage was weaker, suggesting that they might have originated from a native and more primitive group of existing *Globodera*.

## Materials and methods

### Nematodes

*Gv* used in this experiment was collected from the potato root system and soil in the potato fields in Hezhang County, Guizhou Province, China, and was separated, identified by the Plant Nematode Laboratory of South China Agricultural University. It was preserved and propagated in potato root. According to the method of Zasada et al.^56^, sodium orthovanadate solution (0.1 mg/mL) was prepared as an inorganic salt incubator for hatching the second-stage juveniles (J2s). The cysts were sterilised with 0.5% sodium hypochlorite solution for 3 min, then washed with sterile water 5 times, soaked in sterile water for 2 days, then transferred into sodium orthovanadate solution and incubated in a constant temperature incubator at 22 °C. The hatched J2s were collected every 2 days into a 1.5-mL centrifuge tube, disinfected with 0.15% sodium hypochlorite solution then washed with sterile water several times. Afterwards, they were suspended and stored at − 80 °C for subsequent use.

### Molecular identification of nematodes

To ensure that all materials used in the experiment were derived from the spheroid cysts of the same species, three J2s hatched from each cyst were collected using a pick needle under a microscope for PCR identification of a single nematode. The DNA from a single nematode was extracted using the method described by Xu et al.^61^ for ITS sequence amplification using rDNAF/rDNAR primers^62^, and the products were sent to Sangon Biotechnology Co., Ltd. (Shanghai, China) for sequencing. Sequence similarity analysis was performed using MEGA 11 software, and it was determined that all cysts belonged to the same species.

### High-throughput sequencing of mitochondrial DNA, mtDNA assembly and annotation

A total of thirty thousand J2s, hatched and isolated from cysts, were used to for the total DNA extraction and obtained 0.5 μg DNA with a concentration ≥ 20 ng/μl for high-throughput sequencing of mitochondrial DNA. The total DNA and mtDNA of *Gv* were sequenced and assembled respectively using an Illumina NovaSeq and Nanopore, which were entrusted to Huitong Biotechnology Co., Ltd. (Shenzhen, China). When total DNA was sequenced using an Illumina NovaSeq, the original image data were converted into sequence data via Base Calling. Some low-quality data were screened using the fastqc online service website: http://www.bioinformatics.babraham.ac.uk/projects/fastqc/, which comprised the following steps: removing an adapter sequence in the reads, shearing and removing a base containing a non-ATGC at the 5′ end, trimming and sequencing the reads with a mass value less than Q20, removing the reads with the proportion of N reaching 10%, deleting and removing the adapter and small fragments with a mass trimmed length of less than 50 bp. Nanopore sequencing of the total DNA was completed according to the standard protocol provided by Oxford Nanopore Technologies, which comprised the following steps: purity, concentration, integrity detection of genomic DNA by Nanopore, Qubit, and 0.35% agarose gel electrophoresis; recovery of large fragments of DNA by the BluePippin automatic nucleic acid recovery system; and library construction using the SQK-LSK109 connecting kit.

De novo assembly was used to complete the initial concatenation with SPAdes 3.11.0, and the careful mode was used to self-correct the hammer sequence and spliced postback correction sequence. The mitochondrial genome and protein-encoding gene sequences of related species (*G*. *rostochiensis* and *G*. *pallida*) published in NCBI were used as reference comparison sequences for BLASTN and ExONERATE alignments, with the alignment thresholds set as E-value 1e^−10^ and protein similarity threshold of 70%. Sequences with longer matching sequences and higher similarity were screened from the comparison results as target sequences. The PRICE and MITObim software were used to iteratively extend, merge and splice the collected fragmented target sequences. The number of iterations was set to 50. For the iterative splicing, bowtie2 was used to post back the original sequenced reads, and the paired reads were screened. SPAdes was used for re-splicing, and VelvetOptimiser was used to optimise the splicing results. After splicing, sequence integration was performed to complete the assembly of the annular genome.

The sequences were aligned with the NCBI NT library to determine the species annotation gene types and lengths of the near-source sequences. Gene annotation was performed on the Mitos (http://mitos.bioinf.uni-leipzig.de/index.py) and MFannot online sites (https://megasun.bch.umontereal.ca/cgi-bin/MFANNOT/MFANNOTINTERFACE.pl). The genetic codes were 05-InVertebrate and 5-Invertebrate Mitochondrial. The initiation and termination codons were manually corrected for the annotated genes using two annotation methods. The corrected annotated genes were aligned using the NCBI NT library for secondary corrections.

### Bioinformatic analysis of mtDNA

The structural diagram of the scmtDNAs of *Gv* were drawn using graphics softwares, such as Photoshop CS2 9.0 and CorelDraw 12chs. The nucleotide composition of the mitochondrial genome and codon usage of the protein gene were determined using MEGA 11 and DNA star softwares, respectively. The nucleotide composition bias of the gene was calculated by DNA star software and formula, where AT skew value (AT skew) = [A−T]/[A + T], and GC skew value (GC skew) = [G−C]/[G + C]^42^. The effective number of codons (ENC) for mitochondrial protein genes was calculated and analysed using Clustalx 1.83 software and EMBOSS Explorer online (https://www.bioinformatics.nl/emboss-explorer/)^63^. The similarity of different sequences was compared using Clustalx 1.83 and DNA star.

### PCR verification of sequencing results

To verify the results of high-throughput sequencing, two specific segments of approximately 3 kb, referring to the results of high-throughput sequencing, were selected from each scmtDNA, and primers were designed (S1 Table.). Total DNA was used as a template for PCR amplification, and the products were sequenced and analysed.

### Phylogenetic analysis

Owing to the large nucleic acid sequences of the mtDNAs of *Globodera* and the variable arrangement of each gene in different species, a relatively well-conserved gene (*COX1* gene) and a variable gene (*ND1* gene) among species were selected to construct phylogenetic trees. Primers of *COX1* gene and *ND1* gene were designed according to the sequencing results, and the DNA of *Gv* was used as a template for *COX1* gene and *ND1* gene PCR amplification, respectively. The PCR products were sequenced and analysed. The nucleotide composition of the sequence was analysed using EditSeq software, and the A + T content was calculated. *COX1* gene sequences of 26 nematode species (or populations) (S2 Table.) and *ND1* gene sequences of 18 nematode species (or populations) (S3 Table.) published in the NCBI were used as reference sequences to construct phylogenetic trees. The sequences were aligned by MEGA 11 software with default parameters, and the conserved region was selected and the redundancy were removed by Gblocks 0.91b software with half gap. The parameter values of each model were calculated by PAUP software, and the best model setting parameters were determined by MrModel Test software. Phylogenetic analyses were performed using Bayesian Inference (BI) approach^64^ with the operation basis of AIC (Akaike information criterion). The BI approach was performed by MrBayes v3.2.1 software and the specific parameters were as follows: Four independent Markov Chain Monte Carlo (MCMC) models were used, running for two million generations, sampling once every one hundred generations. After discarding the first 25% aged samples, the parameters were summarized, and the remaining samples were used for checking Bayesian posterior probability (PP). When the frequency average standard deviation was less than 0.01, the operation could be stopped.

### Ethics statement

No specific permissions were required for the nematodes used in this study, and these nematodes were plant pests and not protected by the government. This research is carried out in accordance with relevant designated guidelines and regulations. Manuscripts complied with the Animal Research: Reporting of In Vivo Experiments (ARRIVE) guidelines.

### Supplementary Information

Supplementary Information.

## Data Availability

All data generated or analysed during this study are included in its supplementary information files. The original data has been uploaded to the National Center for Biotechnology Information database (GenBank: PP407208-PP407212).

## References

[1] Hockland S, Niere B, Grenier E, Blok V, Phillips M, den Loes N, Anthoine G, Pickup J, Viaene N (2012). An evaluation of the implications of virulence in non-European populations of *Globodera pallida* and *G. rostochiensis* for potato cultivation in Europe. Nematology.

[2] Handoo ZA, Subbotin SA, Perry RN, Moens M, Jones JT (2018). Taxonomy, identification and principal species. Cyst Nematodes.

[3] Urwin PE, Troth KM, Zubko EI, Atkinson HJ (2001). Effective transgenic resistance to *Globodera pallida* in potato field trials. Mol. Breed..

[4] Bates JA, Taylor E, Gans PT, Thomas JE (2010). Determination of relative proportions of *Globodera* species in mixed populations of potato cyst nematodes using PCR product melting peak analysis. Mol. Plant Pathol..

[5] Trudgill DL (1986). Yield losses caused by potato cyst nematodes: a review of the current position in Britain and prospects for improvements. Ann. Appl. Biol..

[6] Urwin PE, Green J, Atkinson HJ (2000). Resistance to *Globodera* spp. in transgenic *Solanum tuberosum* cv. Désirée that express proteinase inhibitors. Aspects Appl. Biol..

[7] Brodie B (2001). Biology and distribution of potato cyst nematodes in North America and their economic impact on potato production. Photo Assoc. Am..

[8] Nicol JM, Turner SJ, Coyne DL, den Nijs L, Hockland S, Tahna Maafi Z, Jones J, Gheysen G, Fenoll C (2011). Current nematode threats to world agriculture. Genomics and Molecular Genetics of Plant-Nematode Interactions.

[9] Jatala P, Bridge J, Luc M, Sikora A, Bridge J (1990). Nematode parasites of root and tuber crops. Plant Parasitic Nematodes in Subtropical and Tropical Agriculture.

[10] Pickup J, Roberts A, den Nijs L, Perry RN, Moens M, Jones JT (2018). Quarantine, distribution patterns and sampling. Cyst Nematodes.

[11] Karnkowska A (2016). A eukaryote without a mitochondrial organelle. Curr. Biol..

[12] Breton S, Ghiselli F, Guerra D, Stewart DT, Passamonti M (2014). A resourceful genome: Updating the functional repertoire and evolutionary role of animal mitochondrial DNAs. Trends Genet..

[13] Le TH, Blair D, Mcmanus DP (2002). Mitochondrial genomes of parasitic flatworms. Trends Parasitol..

[14] Piganeau G, Eyre-Walker A (2009). Evidence for variation in the effective population size of animal mitochondrial DNA. PLoS ONE.

[15] Blouin MS (1998). Mitochondrial DNA diversity in nematodes. J. Helminthol..

[16] Armstrong MR, Blok VC, Phillips MS (2000). A multipartite mitochondrial genome in the potato cyst nematode *Globodera pallida*. Genetics.

[17] He Y, Jones J, Armstrong M, Lamberti F, Moens M (2005). The mitochondrial genome of *Xiphinema americanum* sensu stricto (Nematoda: Enoplea): Considerable economization in the length and structural features of encoded genes. J. Mol. Evolut..

[18] Gibson T (2007). The mitochondrial subgenomes of the nematode *Globodera pallida* are mosaics: Evidence of recombination in an animal mitochondrial genome. J. Mol. Evolut..

[19] Gibson T, Blok VC, Dowton M (2007). Sequence and characterization of six mitochondrial subgenomes from *Globodera rostochiensis*: multipartite structure is conserved among close nematode relatives. J. Mol. Evolut..

[20] Gibson T (2011). The mitochondrial genome of the soybean cyst nematode *Heterodera glycines*. Genome.

[21] Sultana T, Han H, Park JK (2013). Comparison of complete mitochondrial genomes of pine wilt nematode *Bursaphelenchus xylophilus* and *Bursaphelenchus mucronatus* (Nematoda: Aphelenchoidea) and development of a molecular tool for species identification. Gene.

[22] Humphreys-Pereira DA, Elling AA (2014). Mitochondrial genomes of *Meloidogyne chitwoodi* and *M. incognita* (Nematoda: Tylenchina): Comparative analysis, gene order and phylogenetic relationships with other nematodes. Mol. Biochem. Parasitol..

[23] Sun L, Zhuo K, Lin B, Wang H, Liao J, Philippe CS (2014). The complete mitochondrial genome of *Meloidogyne graminicola* (Tylenchina): A unique gene arrangement and its phylogenetic implications. PLoS ONE.

[24] Sun LH (2014). The complete mitochondrial genome of *Aphelenchoides besseyi* (Nematoda: Aphelenchoididae), the first sequenced representative of the subfamily Aphelenchoidinae. Nematology.

[25] Humphreys-Pereira DA, Elling AA (2015). Mitochondrial genome plasticity among species of the nematode genus *Meloidogyne* (Nematoda: Tylenchina). Gene.

[26] Phillips WS (2016). The mitochondrial genome of *Globodera ellingtonae* is composed of two circles with segregated gene content and differential copy numbers. BMC Genom..

[27] Ma X, Agudelo P, Richards VP, Baeza JA (2020). The complete mitochondrial genome of the Columbia lance nematode, *Hoplolaimus columbus*, a major agricultural pathogen in North America. Parasites Vectors.

[28] Xue Q, Du HR, Xue HY, Wang YH, Wang X, Li HM (2021). Mitochondrial genome and phylogeny of *Aphelenchoides medicagus*. Biotechnol. Bull..

[29] Sloan DB (2013). One ring to rule them all? Genome sequencing provides new insights into the ‘master circle’ model of plant mitochondrial DNA structure. New Phytol..

[30] Mindell DP, Dimcheff SDE (1998). Multiple independent origins of mitochondrial gene order in birds. Proc. Natl. Acad. Sci. U. S. A..

[31] Riepsamen AH, Blok VC, Phillips M, Gibson T, Dowton M (2008). Poly(T) variation within mitochondrial protein-coding genes in *Globodera* (Nematoda: Heteroderidae). J. Mol. Evolut..

[32] Hebert P, Cywinska A, Ball SL, Dewaardl JR (2003). Biological identifications through DNA barcodes. Proc. R. Soc. B Biol. Sci..

[33] Morise H, Miyazaki E, Yoshimitsu S, Eki T (2012). Profiling nematode communities in unmanaged flowerbed and agricultural field soils in Japan by DNA barcode sequencing. PLoS ONE.

[34] Bogale M, Baniya A, DiGennaro P (2020). Nematode identification techniques and recent advances. Plants.

[35] Subbotin SA, Franco J, Knoetze R, Roubtsova TV, Vera DP (2019). DNA barcoding, phylogeny and phylogeography of the cyst nematode species from the genus *Globodera* (Tylenchida: Heteroderidae). Nematology.

[36] Ohki T (2018). Molecular characterization of *Globodera pallida* found in Japan using ribosomal DNA and mitochondrial cytochrome b gene sequences. J. General Plant Pathol..

[37] Picard D, Sempere T, Plantard O (2007). A northward colonisationof the Andes by the potato cyst nematode during geologicaltimes suggests multiple host-shifts from wild to cultivated potatoes. Mol. Phylogenet. Evolut..

[38] Plantard O, Picard D, Valette S, Scurrah M, Grenier E, Mugniery D (2008). Origin and genetic diversity of Western European populations of the potato cyst nematode (*Globodera pallida*) inferred from mitochondrial sequences and microsatellite loci. Mol. Ecol..

[39] Jiang R (2022). First record of the golden potato nematode *Globodera rostochiensis* in Yunnan and Sichuan provinces of China. J. Integr. Agric..

[40] Gu JF, Shao BL, Fang YW, Ma XX, Li XY, Zheng JW (2022). Morphological and molecular identification of potato cyst nematode from Sichuan. Fujian J. Agric. Sci..

[41] Peng D (2022). First detection of the potato cyst nematode (*Globodera rostochiensis*) in a major potato production region of China. Plant Dis..

[42] Xu C (2023). Morphological and molecular characterization, including parasitic and pathogenic studies of a new spherical cyst nematode species, *Globodera vulgaris* n. Sp. (Nematoda: Heteroderidae), associated with potatoes in China. Phytopathology.

[43] Perna NT, Kocher TD (1995). Patterns of nucleotide composition at fourfold degenerate sites of animal mitochondrial genomes. J. Mol. Evolut..

[44] Zhang Z, Dai W, Dai D (2013). Synonymous codon usage in TTSuV2: Analysis and comparison with TTSuV1. PLoS ONE.

[45] Liu G, Shao R, Li J, Zhou D, Li H, Zhu X (2013). The complete mitochondrial genomes of three parasitic nematodes of birds: A unique gene order and insights into nematode phylogeny. BMC Genom..

[46] Cedergren RJ, Larue B, Sankoff D, Lapalme G, Grosjean H (1980). Convergence and minimal mutation criteria for evaluating early events in tRNA evolution. Proc. Natl. Acad. Sci. U. S. A..

[47] Lavrov DV, Lang BF (2005). Transfer RNA gene recruitment in mitochondrial DNA. Trends Genet..

[48] Wang X, Lavrov DV (2010). Gene recruitment - A common mechanism in the evolution of transfer RNA gene families. Gene..

[49] Lavrov DV, Brown WM (2001). Trichinella spiralis mtDNA: A nematode mitochondrial genome that encodes a putative *ATP8* and normally structured tRNAs and has a gene arrangement relatable to those of coelomate metazoans. Genetics.

[50] Hu M, Chilton NB, Gasser RB (2003). The mitochondrial genomics of parasitic nematodes of socio-economic importance: Recent progress, and implications for population. Adv. Parasitol..

[51] Wolstenholme DR (1992). Animal mitochondrial DNA: Structure and evolution. Int. Rev. Cytol..

[52] Bernt M, Bleidorn C, Braband A, Dambach J, Donath A, Fritzsch G, Golombek A, Hadrys H, Juhling F, Meusemann K, Middendorf M, Misof B, Perseke M, Podsiadlowski L, von Reumont B, Schierwater B, Schlegel M, Schr€odl M, Simon S, Stadler PF, St€oger I, Struck TH (2013). A comprehensive analysis of bilaterian mitochondrial genomes and phylogeny. Mol. Phylogenet. Evolut..

[53] Cameron SL, Johnson KP, Whiting MF (2007). The mitochondrial genome of the screamer louse *Bothriometopus* (Phthiraptera: Ischnocera): Effects of extensive gene rearrangements on the evolution of the genome. J. Mol. Evolut..

[54] Wei SJ, Shi M, Chen XX, Sharkey MJ, van Achterberg C, Ye GY, He JH (2010). New views on strand asymmetry in insect mitochondrial genomes. PLoS ONE.

[55] Zhang J, Kumar S (1997). Detection of convergent and parallel evolution at the amino acid sequence level. Mol. Biol. Evolut..

[56] Romero H, Zavala A, Musto H (2000). Codon usage in *Chlamydia trachomatis* is the result of strand-specific mutational biases and a complex pattern of selective forces. Nucleic Acids Res..

[57] Liang FF (2010). Influencing factors of codon bias and its research significance. Anim Husband. Feed Sci..

[58] Lü H (2005). Analysis of synonymous codon usage bias in *Chlamydia*. Acta Biochim. Biophys. Sin..

[59] Sharp PM, Emery LR, Zeng K (2010). Forces that influence the evolution of codon bias. Philos. Trans. R. Soc. B Biol. Sci..

[60] Zasada IA, Peetz A, Wade N, Navarre RA, Inghamet RE (2013). Host status of different potato (*Solanum tuberosum*) varieties and hatching in root diffusates of *Globodera ellingtonae*. J. Nematol..

[61] Xu CL, Zhao CB, Ding S, Zhang JF, Xie H (2016). Amodified crude DNA preparation for direct PCR reaction of single plant-parasitic nematodes. Nematology.

[62] Vrain TC, Wakarchuk DA, Lévesque AC, Hamilton RI (1992). Intraspecific rDNA restriction fragment length polymorphism in the *Xiphinema americanum* group. Fund. Appl. Nematol..

[63] Barbhuiya PA, Uddin A, Chakraborty S (2021). Codon usage pattern and evolutionary forces of mitochondrial ND genes among orders of class Amphibia. J. Cell Physiol..

[64] Ronquist F (2012). Mrbayes 3.2: Efficient bayesian phylogenetic inference and model choice across a large model space. Syst. Biol..

