# Supplementary material for: The completed mitochondrial genomes of Globodera vulgaris reveals new insights into the genus Globodera phylogeny
Source: Sci Rep. 2024 Mar 27;14:7253. doi: 10.1038/s41598-024-57736-1 (PMC10973440; doi:10.1038/s41598-024-57736-1)
Supplement: Supplementary file 1 — Supplementary Information. [file 41598_2024_57736_MOESM1_ESM.docx]

Table S1 Primers used in this study

| Primer name | Sequence (5' to 3') | Amplification region | Primer use |
| --- | --- | --- | --- |
| HY-mt1-F1 | CCCTCTACCACTAAACCCTC | scmtDNA-I (4196-7291) | Diagnosis of the mitochondrial genome |
| HY-mt1-R1 | GGAACGGAAGAACGACTACG |  |  |
| HY-mt1-F2 | CGGAGAATGTCAAAGAAACC | scmtDNA-I (310-3193) |  |
| HY-mt1-R2 | TTAGCGTGATTGGTCGAAGG |  |  |
| HY-mt2-F1 | GAAACCAAACATCAAACAGG | scmtDNA-II (3651-6713) |  |
| HY-mt2-R1 | ATAACAGGACTCAGACCACC |  |  |
| HY-mt2-F2 | TCGTTAGGTCGGACATTTGG | scmtDNA-II (86-3046) |  |
| HY-mt2-R2 | AATGGTGGCTTTGAGAACAC |  |  |
| HY-mt3-F1 | ATACATGCACGTAGGAACGG | scmtDNA-III (2906-5947) |  |
| HY-mt3-R1 | GAATTGAATAAAGGCAGACC |  |  |
| HY-mt3-F2 | AGGGATAGAGGATATGGAAG | scmtDNA-III (6105-9110) |  |
| HY-mt3-R2 | AAGACCACCCACCAAAGATC |  |  |
| HY-mt4-F1 | TGTTTACCATTGGAGGATTG | scmtDNA-IV (5376-8391) |  |
| HY-mt4-R1 | ACTCTGTTAGCACCTTAGCC |  |  |
| HY-mt4-F2 | GAAGAACGACTACGAGAGTAG | scmtDNA-IV (358-3300) |  |
| HY-mt4-R2 | GTATTGTCCAAGTAAACCCAC |  |  |
| HY-mt5-F2 | ATCCTTAACCTTACCCGAAC | scmtDNA-V (5029-8015) |  |
| HY-mt5-R2 | ATAACAGGACTCAGACCAC |  |  |
| HY-mt1-F2 | GAGCCAAGAACTCTGAACG | scmtDNA-V (1258-4172) |  |
| HY-mt1-R2 | GGTATTACTGGTGGTTGAG |  |  |
| rDNAF | GTTTCCGTAGGTGAACCTGC | ITS | ITS region amplification |
| rDNAR | ATATGCTTAAGTTCAGCGGGT |  |  |
| CXF | CCATTAAAATTACGATCAACTA | pCOX1 | COX1 region amplification |
| CXR | CCTAAAACATAATGAAAATGAGC |  |  |
| ND1mt1F | GGGCAGCGTTTAATATTTGA | ND1 in scmtDNA-I | ND1 region amplification |
| ND1mt1R | AATAAGGACTTGAGAGGAAGG |  |  |
| ND1mt5F | ACCAGTCCTAAACCAAGAAA | ND1 in scmtDNA-V |  |
| ND1mt5R | GGGTTAATTAGTGGGTATGTTC |  |  |

Table S2 Sequence information of *COX1* gene in reference nematode mitochondrial

| species | length/bp | origin | Genbank |
| --- | --- | --- | --- |
| *Globodera agulhasensis* | 443 | South Africa | MN095891 |
| *G. artemisiae* | 443 | Germany | MN095894 |
| *G. artemisiae* | 443 | Russia | MN095892 |
| *G. capensis* | 443 | South Africa | MN095880 |
| *G. ellingtonae* | 1509 | USA | KU726971 |
| *G. Mexicana* | 443 | Mexico | MN095877 |
| *G. millefolii* | 443 | Estonia | MN095889 |
| *G. pallida* | 443 | Spain | MN095897 |
| *G. pallida* | 443 | Peru | MN095916 |
| *G. pallida* | 443 | Bolivia | MN095923 |
| *G. pallida* | 1389 | British | DQ631912 |
| *G. rostochiensis* | 443 | Germany | MN095979 |
| *G. rostochiensis* | 443 | Bolivia1 | MN095980 |
| *G. rostochiensis* | 443 | Bolivia2 | MN095982 |
| *G. sandveldensis* | 443 | South Africa | MN095888 |
| *G. tabacum* | 443 | Bolivia | MN096053 |
| *G. tabacum* | 443 | Argentina | MN096065 |
| *G. tabacum* | 443 | Italy | MN096067 |
| *G. tabacum* | 443 | France | MN096044 |
| *G. tabacum* | 443 | USA | MN096041 |
| *G. zelandica* | 443 | New Zealand | MN095887 |
| *Heterodera glycines* | 1524 | USA | HM640930 |
| *Meloidogyne chitwood* | 1563 | California | KJ476150 |
| *M. incognit* | 1522 | Costa Rica | KJ476151 |
| *Pratylenchus vulnus* | 1533 | USA | NC_020434 |
| *Radopholus similis* | 1557 | Belgium | NC_013253 |
| *Trichuris ovis* | 1545 | China | NC_018597 |

Table S3 Sequence information of *ND1* gene in reference nematode mitochondrial

| species | length/bp | origin | Genbank |
| --- | --- | --- | --- |
| *Globodera rostochiensis* | 657 | Australia | EF462977 |
| *G. ellingtonae* | 843 | USA | KU726972 |
| *G. pallida* | 830 | Australia | DQ631911 |
| *G. pallida* | 864 | USA | AJ249395 |
| *Heterodera glycines* | 834 | Australia | HM640930 |
| *Meloidogyne chitwood* | 841 | USA | KJ476150 |
| *M. incognit* | 850 | USA | KJ476151 |
| *M. arenaria* | 850 | USA | NC_026554 |
| *M. javanica* | 850 | USA | NC_026556 |
| *M. enterolobii* | 850 | USA | NC_026555 |
| *M. graminicola* | 885 | China | NC_056772 |
| *Pratylenchus vulnus* | 873 | South Korea | NC_020434 |
| *Radopholus similis* | 861 | Belgium | NC_013253 |
| *Bursaphelenchus mucronatus* | 876 | South Korea | NC_021120 |
| *B. xylophilus* | 873 | South Korea | NC_023208 |
| *Aphelenchoides besseyi* | 882 | China | NC_025291 |
| *A. medicagus* | 873 | China | NC_061210 |
| *Trichuris ovis* | 900 | China | NC_018597 |

**Figure S1. PCR amplification electrophoresis map of a specific fragment of the subgenomic mitochondrial circles (scmtDNA) in *Globodera vulgaris***

**M**

**1**

**2**

**3**

**4**

**5**

**6**

**7**

**8**

**9**

**10**

**5000 bp**

**3000 bp**

**2000 bp**

**1000 bp**

**7500 bp**

**500 bp**

**250 bp**

**100 bp**


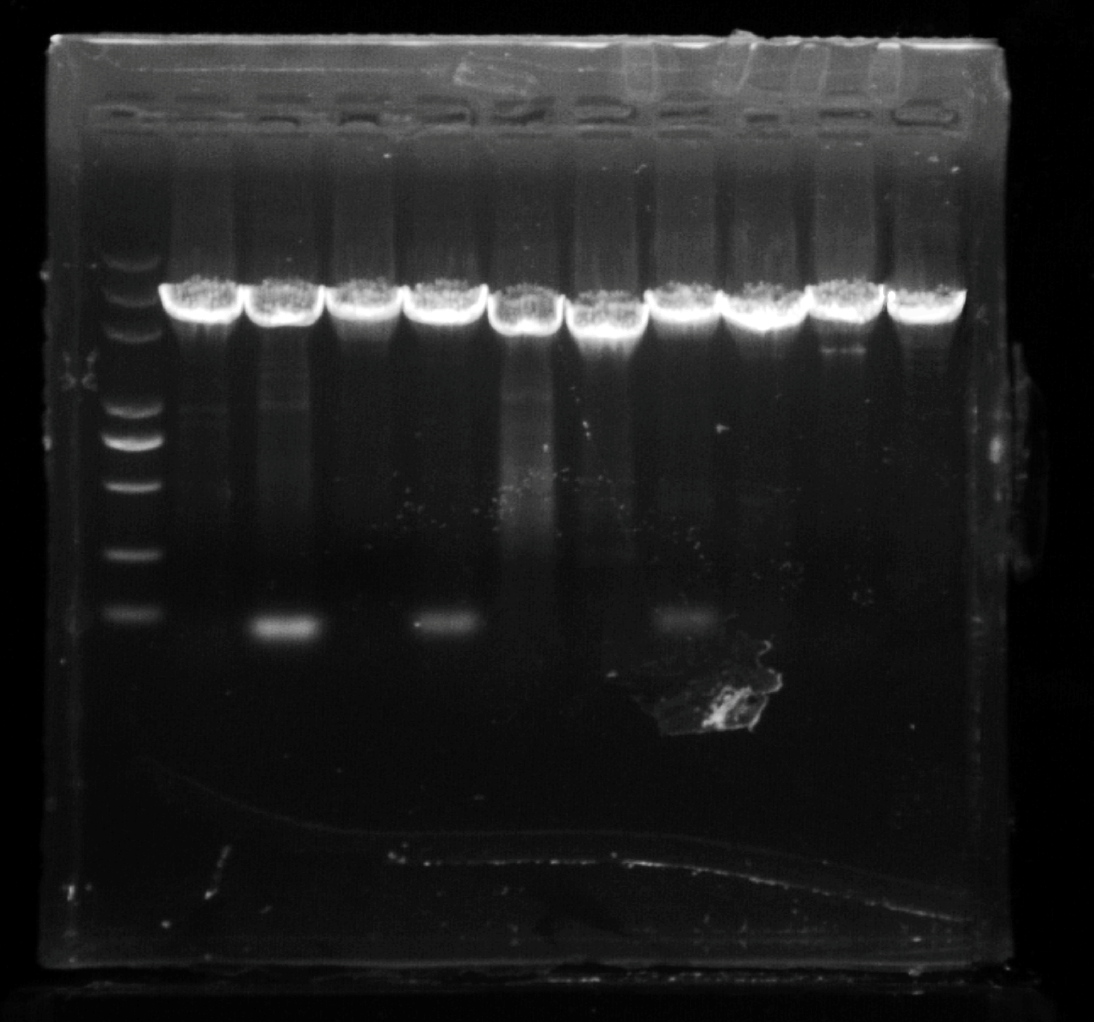


M: DL5000 marker; 1 and 6: the amplification results of two specific primers, HY-mt1-F1/HY-mt1-R1 and HY-mt1-F2/HY-mt1-R2, of scmtDNA-I, respectively; 2 and 7: the amplification results of two specific primers, HY-mt2-F1/HY-mt2-R1 and HY-mt2-F2/HY-mt2-R2, of scmtDNA-II, respectively; 3 and 8: the amplification results of two specific primers, HY-mt3-F1/HY-mt3-R1and HY-mt3-F2/HY-mt3-R2, of scmtDNA-III, respectively; 4 and 9: the amplification results of two specific primers, HY-mt4-F1/HY-mt4-R1and HY-mt4-F2/HY-mt4-R2, of scmtDNA-IV, respectively; 5 and 10: the amplification results of two specific primers, HY-mt5-F1/HY-mt5-R1and HY-mt5-F2/HY-mt5-R2, of scmtDNA-V, respectively.
